# Simultaneously Promoting Proton Conductivity and Mechanical Stability of SPEEK Membrane by Incorporating Porous g–C_3_N_4_

**DOI:** 10.3390/membranes15070194

**Published:** 2025-06-29

**Authors:** Xiaoyao Wang, Benbing Shi

**Affiliations:** 1 Guangdong Water Co., Ltd., Shenzhen 518021, China; 2 School of Environment, Harbin Institute of Technology, Harbin 150090, China; 3 College of Food Quality and Engineering, Nanning University, Nanning 530000, China; 4 College of Environmental Science and Engineering, Nankai University, Tianjin 300110, China

**Keywords:** proton exchange membrane, sulfonated poly(ether ether ketone), g–C_3_N_4_

## Abstract

Proton exchange membranes are widely used in environmentally friendly applications such as fuel cells and electrochemical hydrogen compression. In these applications, an ideal proton exchange membrane should have both excellent proton conductivity and mechanical strength. Polymer proton exchange membranes, such as sulfonated poly(ether ether ketone) (SPEEK) membranes with high ion exchange capacity, can lead to higher proton conductivity. However, the ionic groups may reduce the interaction between polymer segments, lower the membrane’s mechanical strength, and even cause it to dissolve in water as the temperature exceeds 55 °C. The porous graphitic C_3_N_4_ (Pg–C_3_N_4_) nanosheet is an important two–dimensional polymeric carbon–based material and has a high content of –NH_2_ and –NH– groups, which can interact with the sulfonic acid groups in the sulfonated SPEEK polymer, form a more continuous proton transfer channel, and inhibit the movement of the polymer segment, leading to higher proton conductivity and mechanical strength. In this study, we found that a SPEEK membrane containing 3% Pg–C_3_N_4_ nanosheets achieves the optimized proton conductivity of 138 mS/cm (80 °C and 100% RH) and a mechanical strength of 74.1 MPa, improving both proton conductivity and mechanical strength by over 50% compared to the SPEEK membrane.

## 1. Introduction

As a clean and efficient alternative energy source, proton exchange membrane fuel cells are an ideal choice for solving energy crises and environmental pollution problems [1]. As the core component of proton exchange membrane fuel cells, the proton exchange membrane should possess both superior proton conductivity and structural stability [2,3,4,5]. Poly(ether ether ketone) (PEEK) is highly favored in laboratory research and commercial applications owing to its excellent mechanical strength and chemical stability [6]. Sulfonic acid functional groups can be introduced into the aromatic main chain of PEEK through electrophilic substitution reactions to form sulfonated poly(ether ether ketone) (SPEEK) and enhance proton conductivity. Based on the Nernst–Einstein equation for ion conduction, σ = Dnq^2^/KT, proton conductivity is positively correlated with the number of proton donor groups in the membrane. Increasing the number of proton donor exchange groups, such as sulfonate groups, can significantly increase the proton conductivity of the membrane. However, the interaction between polymer chains is reduced, and the membrane swells excessively or even dissolves in water [7]. For example, Yu et al. [8] prepared SPEEK membranes with different sulfonation degrees, and found that when the sulfonation degree increased from 57% to 87%, the membrane swelling ratio increased significantly from 4.2 to 27.7%, and the membrane breaking strength decreased from 49.9 MPa to 24.1 MPa. Therefore, there is a trade–off effect between the proton conductivity and structural stability of the SPEEK membrane, which has become a bottleneck problem restricting the research and development of high–conductivity and highly stable proton exchange membranes. Organic and inorganic nanomaterials, such as metal–organic frameworks [9], covalent organic frameworks [10], graphene oxide [11], etc., have been used to improve the stability and proton conductivity of membranes. For example, Zhang et al. [9] used Fe–MIL–101–NH_2_ as an additive to prepare SPEEK/MOF composite membranes. The proton conductivity of the membrane containing 2% Fe–MIL–101–NH_2_ increased from 2.4 to 22.6 mS cm^−1^ (25 °C, 98% RH), and the tensile strength increased from 28 MPa to 59.5 MPa. However, it is still challenging to push the upper limit of ion conductivity and mechanical strength of the SPEEK membrane.

Graphitic carbon nitride (g–C_3_N_4_) was an important two–dimensional polymeric carbon–based material with a nanosheet structure composed of carbon and nitrogen atoms [12]. Recent studies have used C_3_N_4_ as an additive to reduce the swelling of SPEEK and improve mechanical strength. The tensile strength of the membrane could reach 51–54 MPa at a low C_3_N_4_ addition amount of 0.5–1% [13,14,15]. Due to the C_3_N_4_ agglomerating in the SPEEK polymer, further increasing the amount of C_3_N_4_ could decrease the membrane proton conductivity and mechanical strength [14,15]. Few–layer porous graphitic C_3_N_4_ (Pg–C_3_N_4_) nanosheets have a higher specific surface area and contain more active sites [12]. It is speculated that Pg–C_3_N_4_ could be introduced to SPEEK polymers in higher amounts without aggregation. The dispersed, high–content C_3_N_4_ can help the composite membrane achieve higher mechanical strength. At the same time, using the Pg–C_3_N_4_ and SPEEK to prepare a composite membrane was expected to create more continuous proton transfer channels and achieve higher proton conductivity.

In this study, we synthesized a SPEEK polymer with high ion exchange capacity (1.85 mmol/g) and blended it with Pg–C_3_N_4_ to prepare the Pg–C_3_N_4_–SPEEK composite membrane. This study explored the effect of 1–4% Pg–C_3_N_4_ addition amounts on the comprehensive properties of the membrane, including water absorption and swelling behavior, mechanical properties, ionic conductivity, and single–cell performance. We found that the SPEEK membrane with 3% Pg–C_3_N_4_ nanosheets achieves an optimum proton conductivity of 138 mS/cm and a mechanical strength of 74.1 MPa, enhancing both proton conductivity and mechanical strength by more than 50% compared to the SPEEK control membrane.

## 2. Materials and Methods

### 2.1. Chemicals

Poly(ether ether ketone) (Victrex^®^PEEK, grade 381G) was purchased from Nanjing Yuanbang Engineering Plastics Co., Ltd., Nanjing, China. Pt/C catalyst and carbon paper were received from Shanghai Hesen Electric Co., Ltd., Shanghai, China. N,N–dimethylacetamide (DMAc, >99%), Melamine, phosphorous acid, were purchased from Shanghai Macklin Biochemical Technology Co., Ltd., Shanghai, China. Glycerol (>99%) and ethanol (>99.5%) were purchased from Shanghai Aladdin Industrial Corporation, Shanghai, China. All commercially available chemicals and solvents were used without further purification.

### 2.2. Preparation of SPEEK

The preparation method of SPEEK was reported in the literature [15], and the specific steps are as follows. Poly(ether ether ketone) (PEEK) particles were dried at 100 °C under vacuum for 24 h to remove moisture. The dried PEEK (20 g) was added to 98% concentrated sulfuric acid (142 mL) and mechanically stirred at 25 °C for 3 h (avoiding excessive rotation speed to generate bubbles). The reaction solution was transferred to a 50 °C constant–emperature water bath and mechanically stirred for 12 h. The reaction product was then slowly poured into deionized water while being mechanically stirred to obtain white filamentous SPEEK. The SPEEK was washed with a large amount of deionized water to pH = 7.0. The obtained SPEEK was naturally dried at room temperature to remove surface moisture. The SPEEK was then dried at 60 °C for one week for the next step use.

### 2.3. Preparation of Pg–C_3_N_4_

Preparation of precursor: According to the literature [12], 1 g of melamine and 1.2 g of phosphoric acid were dissolved in 100 mL of deionized water, heated to 80 °C, and stirred vigorously for 1 h. The solution was subsequently placed in an autoclave lined with polytetrafluoroethylene and heated to 180 °C for 10 h. The resulting mixture was centrifuged and washed multiple times with deionized water. Finally, the precursor was obtained after drying at 60 °C.

**Figure 1:**
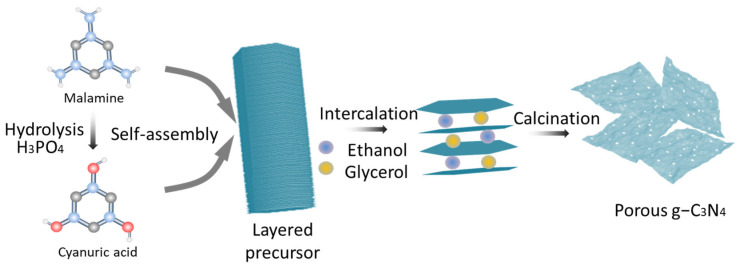
The representation schematic of Pg–C_3_N_4_ preparation.

Preparation of Pg–C_3_N_4_: A total of 0.6 g of the precursor was treated with a mixed aqueous solution comprising 5 mL of glycerol and 15 mL of ethanol at 90 °C for 3 h. The powder was subsequently washed thrice with ethanol and dried at 60 °C. The resultant solid was subjected to heating at 500 °C in a muffle furnace, with a heating rate of 2 °C/min, and maintained at this temperature for 2 h. The sample collected was a few–layer C_3_N_4_. The Pg–C_3_N_4_ preparation representation schematic is shown in Figure 1.

### 2.4. Preparation of SPEEK/Pg–C_3_N_4_ Membranes

**Figure 2:**
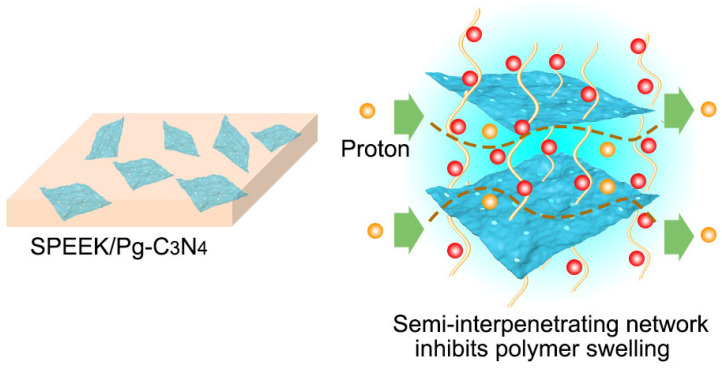
The representation schematic of SPEEK/Pg–C_3_N_4_ membranes.

The SPEEK/Pg–C_3_N_4_ membranes were prepared by the casting method (Figure 2). First, SPEEK (0.6 g) was dissolved into N,N–dimethylformamide (3 mL) under stirring, and 6–24 mg (1–4% wt%) Pg–C_3_N_4_ was dispersed in DMAc (3 mL) and placed under ultrasonic treatment for 12 h. These two kinds of suspensions were then mixed for another 12 h of alternate mechanical stirring to form a uniform solution. The mixed solvent was evaporated at 60–80 °C for 12 h to form a dense membrane structure. After evaporation, the resulting membrane was immersed in 1 mol/L H_2_SO_4_ and further stored in deionized water before use. For comparison, a pristine SPEEK membrane was prepared using the same procedure. Three copies of each membrane were prepared. The composite membranes were designated as SPEEK/PgC_3_N_4_–*X*, where *X* represented the weight percentage of the Pg–C_3_N_4_ relative to SPEEK matrix.

### 2.5. Characterization Methods

#### 2.5.1. Characterizations

The powder X–ray diffraction (PXRD) data were collected on a D/max 2500 v/pc (Rigaku Corporation, Tokyo, Japan) diffractometer; the scanning range was between 3 and 50° and the scanning speed was 5° min^−1^.

The Fourier transform infrared (FT–IR) spectra were recorded on a Vertex 70 (BRUKER, Billerica, MA, USA) spectrometer equipped with a horizontal attenuated transmission accessory.

The membrane cross–section morphology was collected by a field emission scanning electron microscope (Nanosem 430, FEI Company, Hillsboro, USA; and TM4000 Plus II, HITACHI, Tokyo, Japan); the membrane was first immersed in liquid nitrogen and then broken after the membrane became fragile, and the membrane was pasted on the sample table with conductive adhesive. Due to the poor conductivity of polymers, gold was sprayed on for 40 s using a vacuum coating machine to improve the membrane’s conductivity.

Transmission Electron Microscopy (TEM) images were obtained by HRTEM (Tecnai G2 F20, FEI Company, Hillsboro, OR, USA). The nanosheet dispersion was dropped onto the copper grid. After vacuum drying, the copper grid loaded with nanosheets was mounted in the TEM sample rod for testing.

The stress–strain curves of the membranes were obtained by a WDW–02 (Yangzhou Zhongke Co., Ltd., Yangzhou, China) electronic stretching machine with a strain rate of 2 mm min^−1^ under ambient conditions.

Thermogravimetric analysis (TGA) was carried out on a NETZSCH 209F3 (NETZSCH Group, Selb, Germany) thermal analyzer at a heating rate of 10 °C min^−1^ min from 40 to 800 °C and under N_2_ atmosphere.

Differential scanning calorimetry (NETZSCH 204 F1, NETZSCH Group, Selb, Germany) was conducted under a nitrogen atmosphere to detect the glass transition temperature (*T_g_*) of the membranes (heating rate: 10 °C min^−1^, test temperature range: 30–250 °C).

#### 2.5.2. Water Uptake and Swelling Ratio Test

Slice the membrane for testing into 20 mm × 20 mm pieces, then dry it in a vacuum oven at 60 °C for 24 h. Then, weigh the membrane sample mass (*W_dry_*) and measure the membrane area (*A_dry_*). Place the membrane in deionized water to test the water absorption and swelling of the membrane, quickly wipe off the adsorbed water on the membrane surface with filter paper, and quickly weigh the wet membrane mass (*W_wet_*) and measure the wet membrane area (*A_wet_*) at the same time. The water uptake (%) and swelling ratio (%) of the samples were calculated by Equations (1) and (2).

(1) $$Water\;uptake=\frac{{W_{wet}-W}_{dry}}{W_{dry}}$$

(2) $$Swelling\;ratio=\frac{{A_{wet}-A}_{dry}}{A_{dry}}$$

#### 2.5.3. Ion Exchange Capacity (IEC) Test

The IEC values of the membranes were measured by a titration method. The dry samples (0.2 g) were added into NaCl solution (0.01 g/mL, 20 mL) and stirred for 24 h. Then the solutions were titrated using a 0.01 M NaOH standard solution, and phenolphthalein was used as an indicator. The IEC values (mmol g^–1^) of the samples were calculated by Equation (3):

(3) $$IEC=\frac{{C_{NaOH}\times V}_{NaOH}}{W_{dry}}$$

where *C_NaOH_* (mol L^–1^) is the molar concentration of the NaOH solution; *V_NaOH_* (L) is the consumed volume of the NaOH solution during the titration process; and *W_Dry_* (g) is the weight of the dry samples.

#### 2.5.4. Proton Conductivity Measurement

In–plane proton conductivity of the membranes was measured by a two–probe method. The alternating current impedance measurements were performed using an impedance/gain–phase analyzer (PARSTAT4000, Princeton Applied Research, Oak Ridge, TN, USA) in the 1 MHz to 10 Hz range with a signal amplitude of 15 mV. The measurements were conducted at temperatures between 30 °C and 80 °C and 100% RH% inside a thermoshygrostat. The proton conductivities (*σ*, mS cm^−1^) were calculated as follows.

(4) $$\sigma =\frac{l}{AR}$$

where *l* (cm), *A* (cm^2^), and *R* (Ω) are the distance between the electrodes, the cross-section area, and the Ohmic resistance of the membranes, respectively. The cross–sectional area *A* of membranes was calculated by the membrane width times the membrane thickness.

#### 2.5.5. Membrane Electrode Assembly (MEA) Fabrication and Fuel Cell Test

The membrane electrode was prepared by the hot–pressing method. The thickness of the SPEEK and SPEEK/Pg–C_3_N_4_–3 membranes used in this study for fuel cell performance tests was 80 μm. The catalyst (Pt/C 40%), Nafion solution, isopropanol, and deionized water were prepared into a dispersion and coated on the surface of hydrophobic–treated carbon paper. The catalyst content was 0.5 mg·cm^−2^. The catalyst layer and the membrane were then hot–pressed to form a sandwich structure MEA. The MEA was sealed between the two polar plates of a single cell and tested using the NBT–PEM–25W (Ningbo Bate Measurement and Control Technology Co., Ltd., Ningbo, China) system. The conditions for the single–cell performance test include a test temperature of 60 °C, a relative humidity of 100%, a hydrogen flow rate of 100 mL min^−1^, and an oxygen flow rate of 100 mL min^−1^. Back pressures of 1 bar were applied to both the anode and cathode. The fuel cell test adopted a constant current mode with a current density range of 40–1080 mA·cm^−1^ and a step size of 40 mA cm^−1^.

## 3. Results and Discussions

### 3.1. Characterizations of Pg–C_3_N_4_ Nanosheets

**Figure 3:**
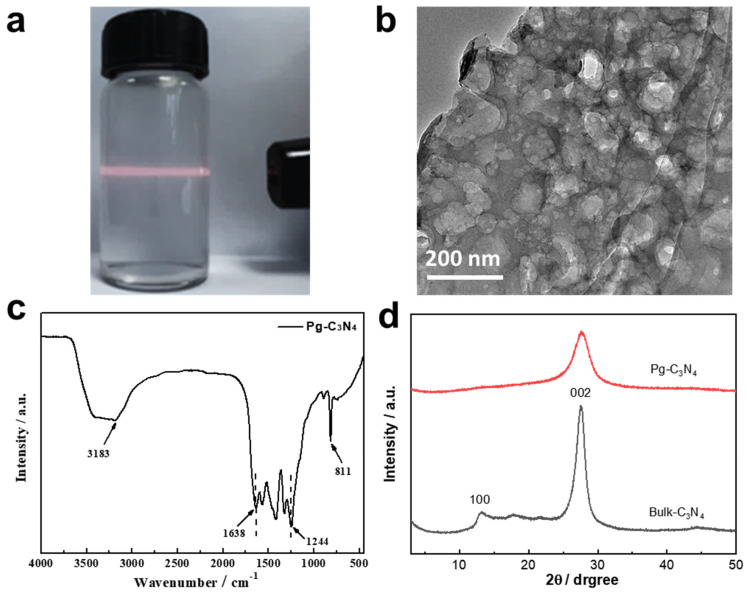
(**a**) Digital photo of Pg–C_3_N_4_ dispersion solution; (**b**) TEM image, (**c**) FTIR spectrum, and (**d**) XRD spectrum of Pg–C_3_N_4_ nanosheets.

Figure 3a shows a digital photograph of the Pg–C_3_N_4_ nanosheet solution. Since the edges of Pg–C_3_N_4_ nanosheets have rich –NH_2_ and –NH groups and good hydrophilicity, Pg–C_3_N_4_ nanosheets can be evenly dispersed in water solution with an obvious Tyndall effect. The SEM, AFM, and TEM images of the Pg–C_3_N_4_ nanosheets are shown in Figure 3b and Figure S1 (Supplementary Materials), which shows that the nanosheets are about 1–3 μm in size and have a porous morphology. The FTIR spectrum of Pg–C_3_N_4_ is shown in Figure 3c; the absorption peak at 810 cm^−^^1^ can be attributed to the breathing mode of the tri–s–triazine ring, and the absorption peak at 1200–1600 cm^−^^1^ can be attributed to the characteristic stretching of the CN heterocyclic, indicating that the short–range structure is well maintained [13]. The broad peak in the range of 3000–3600 cm^−^^1^ can be attributed to the N–H stretching vibration [12]. As shown in the XRD patterns (Figure 3d), the weaker and broader peak at (002) demonstrates the few–layer nature of Pg–C_3_N_4_ nanosheets [12]. The peak at 13.1° stemmed from the in–plane structural packing motif of the (100) lattice plane becoming much weaker in the nanosheets, which may result from in–plane porous structures.

### 3.2. Characterizations of the Membranes

**Figure 4:**
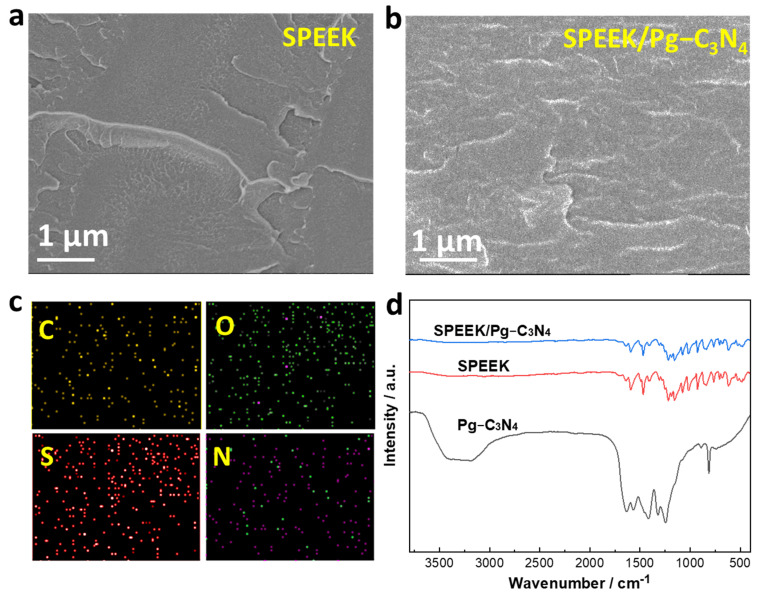
(**a**) SEM cross–section image of the SPEEK membrane; (**b**) SEM cross–section image of the SPEEK/Pg–C_3_N_4_ membrane; (**c**) SEM elemental mapping of cross–section of SPEEK/Pg–C_3_N_4_ membrane; (**d**) FTIR spectrum of SPEEK and SPEEK/Pg–C_3_N_4_ membranes.

The SPEEK/Pg–C_3_N_4_ membranes were prepared by the casting method. First, the Pg–C_3_N_4_ nanosheets and SPEEK are dissolved in a proton–inert polar solvent (N,N–dimethylformamide or dimethyl sulfoxide) to form a uniform solution. The solvent was evaporated at 60–80 °C to form a dense membrane structure (Figure 4a,b). The SPEEK/Pg–C_3_N_4_ membrane cross–section image exhibits a layered structure due to the addition of Pg–C_3_N_4_ nanosheets. The element distribution in the SPEEK/Pg–C_3_N_4_ membrane is shown in Figure 4c, which confirms that the C, O, S, and N elements were evenly distributed in the membrane cross–section and the Pg–C_3_N_4_ nanosheets were evenly distributed in the membrane. The FTIR spectrums of the SPEEK and SPEEK/Pg–C_3_N_4_ membranes are shown in Figure 4d. Due to the electrostatic interaction between SPEEK and Pg–C_3_N_4_ nanosheets, the surface groups of the Pg–C_3_N_4_ nanosheets were covered by SPEEK, and only the absorption peaks of sulfonic acid and other groups owing to the SPEEK polymer were observed.

**Figure 5:**
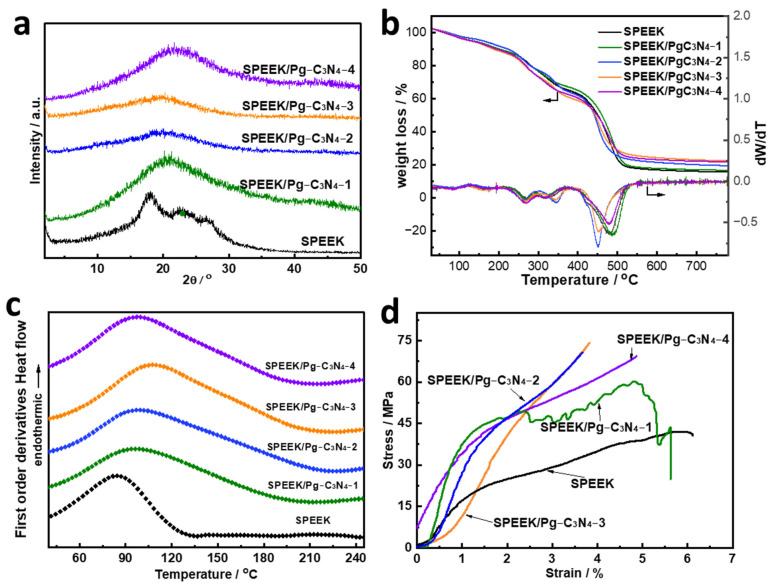
(**a**) XRD patten, (**b**) TGA curves, (**c**) DSC curves, and (**d**) stress–strain curves of SPEEK and SPEEK/Pg–C_3_N_4_ membranes.

The XRD patterns of SPEEK and SPEEK/Pg–C_3_N_4_ with different Pg–C_3_N_4_ additions are shown in Figure 5a. The introduction of Pg–C_3_N_4_ interferes with the arrangement of SPEEK chain segments, which significantly reduces the peak intensity of the XRD pattern of SPEEK. TGA showed that all membranes underwent three distinct degradation steps (Figure 5b). The first degradation below 200 °C was associated with the dehydration and evaporation of residual solvent. The second weight loss in the range of 250–350 °C can be attributed to the degradation of the sulfonic acid groups [15]. The third weight thermal degradation above 400 °C was caused by the decomposition of the SPEEK backbone. The SPEEK/Pg–C_3_N_4_ membranes had a higher degradation temperature because Pg–C_3_N_4_ had excellent thermal stability. The TGA curves showed that all SPEEK/Pg–C_3_N_4_ membranes have sufficiently high thermal stability to be used in PEMFCs.

The DSC curve of the SPEEK and SPEEK/Pg–C_3_N_4_ membranes are shown in Figure 5c. Since the SPEEK synthesized in this study has a high degree of sulfonation and a homogeneous structure, there is no phase change endothermic peak. The blank SPEEK membrane exhibits a clear endothermic peak at 84.9 °C, primarily caused by the loss of water that was adsorbed in the membrane, as indicated by a corresponding weight loss observed in the thermogravimetric curve (Figure 5b) [16,17]. In contrast, the endothermic peak temperature of water molecules increases and the range expands when the SPEEK/Pg–C_3_N_4_ addition amount increases from 0% to 3%. The main causes of this enhancement are that electrostatic interactions prevent SPEEK chain segments from moving and prevent water loss [15]. When the Pg–C_3_N_4_ content increased to 4%, the endothermic peak temperature of water molecules decreased. The decrease in endothermic peak temperature can be explained by the fact that the aggregation of Pg–C_3_N_4_ increased the distance between polymer chains, offsetting the electrostatic interaction and thus enhancing the segmental movement of SPEEK chains [13,15].

The stress–strain curves in Figure 5d could be used to analyze the membranes’ mechanical stability. In comparison to the SPEEK membrane, the composite membranes demonstrated a higher ultimate tensile strength. The SPEEK membrane with 3 wt% Pg–C_3_N_4_ had an ultimate tensile strength of 74.1 MPa, which is 76.8% higher than the blank SPEEK membrane. The improvements in mechanical properties were due to the strong hydrogen bonds and electrical interactions between SPEEK and Pg–C_3_N_4_, which help transfer the mechanical load to the rigid Pg–C_3_N_4_ nanosheets [15]. However, the mechanical stability decreased when the Pg–C_3_N_4_ content increased to 4%, which was primarily ascribed to the more pronounced aggregation of the Pg–C_3_N_4_ nanosheets.

### 3.3. IEC, Proton Conductivity, and Single PEMFC Performance of the Membranes

**Figure 6:**
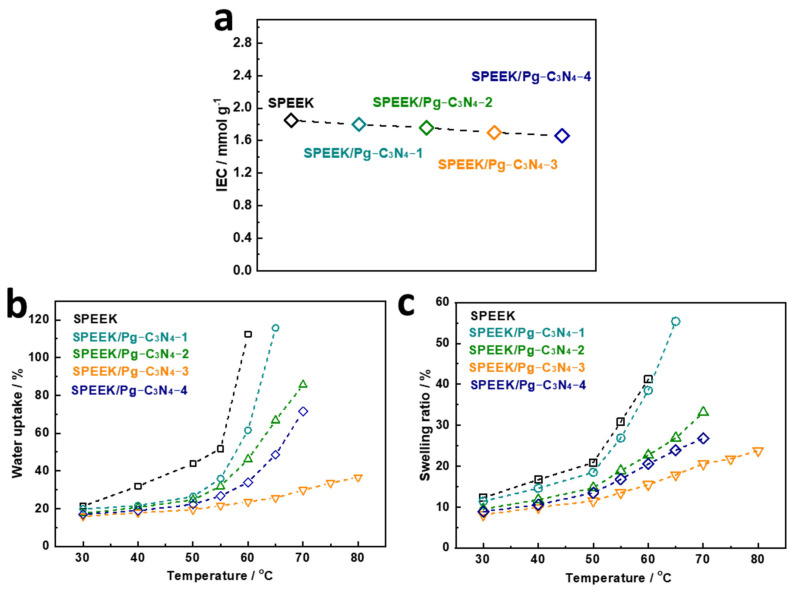
(**a**) IEC, (**b**) water uptake, and (**c**) swelling ratio of SPEEK and SPEEK/Pg–C_3_N_4_ membranes.

The IEC values of SPEEK and SPEEK/Pg–C_3_N_4_ membranes are shown in Figure 6a. The IEC of the SPEEK membrane synthesized in this study was 1.85 mmol/g. The IEC value can be used to calculate the sulfonation degree of the SPEEK polymer [18,19], and the sulfonation degree of the SPEEK membrane synthesized in this study was calculated to be 65.7%. As the amount of Pg–C_3_N_4_ added increases, the IEC value of the composite membrane decreases from 1.85 to 1.66 mmol/g. The proton conductivity can be significantly altered by the water uptake of the proton exchange membrane. Figure 6b,c show the SPEEK and SPEEK/Pg–C_3_N_4_ membranes’ water uptake and swelling ratio. At 30 °C, the SPEEK membrane’s water uptake and swelling ratios were 21.3% and 12.3%, respectively. As the temperature increases, the water uptake and swelling ratio of the membrane gradually increases. When the temperature was higher than 60 °C, the SPEEK polymer was completely dissolved in water. The excessive swelling of the SPEEK membrane at temperatures above 60 °C is mainly attributed to the higher degree of sulfonation and stronger hydrophilicity, which weakens the interaction between SPEEK chain segments. When the Pg–C_3_N_4_ additional amount increases from 0 to 3%, the water uptake and swelling ratio of the composite membrane gradually decrease. The SPEEK/Pg–C_3_N_4_–3 membrane remained stable in 80 °C water, at which the water uptake was 36.6% and the swelling ratio was 23.8%. The decrease in water absorption and swelling of the membrane can be attributed to the electrostatic interaction between Pg–C_3_N_4_ and SPEEK, which inhibits the movement of polymer chain segments [13,15]. However, when the Pg–C_3_N_4_ addition amount increased from 3% to 4%, the water uptake and swelling ratio of the composite membrane increased, and the membrane could dissolve in water at 80 °C. This phenomenon may be attributed to the aggregation of Pg–C_3_N_4_, which increases the distance between polymer chains, reduces the electrostatic interaction, and consequently enhances the segmental movement of SPEEK chains [15]. The above result suggests that the dimensional stability of the composite membranes can be significantly enhanced by strong electrostatic interactions. The optimum state of dimensional stability for the membrane was achieved when the Pg–C_3_N_4_ addition was 3%.

**Figure 7:**
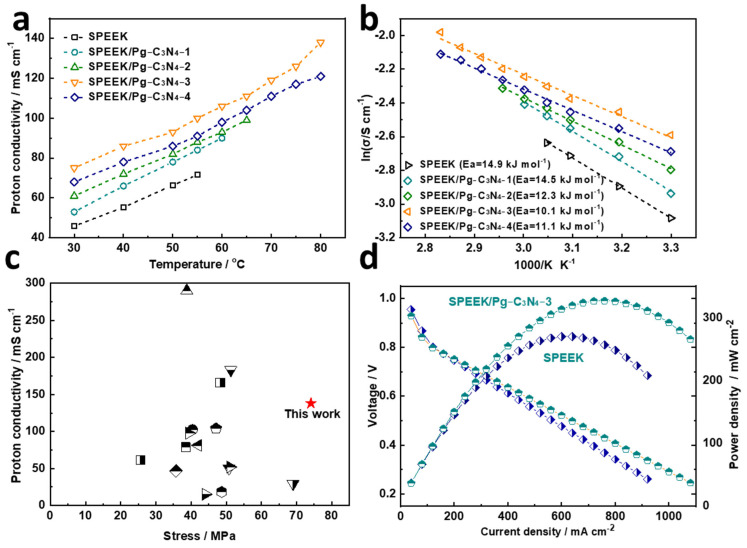
(**a**) Temperature–dependent proton conductivity of the prepared membranes at 100% RH; (**b**) proton–transfer activation energy (*E_a_*) of the prepared membranes; (**c**) proton conductivity and tensile strength of the prepared membranes in comparison with SPEEK–based membranes; (**d**) the fuel cell performance of the SPEEK and SPEEK/Pg–C_3_N_4_–3 membranes.

Proton conductivity is the core performance parameter of proton exchange membranes and plays an important role in the performance of fuel cells. The proton conductivity of the SPEEK and SPEEK/Pg–C_3_N_4_ membranes at 100% RH and 30–80 °C are shown in Figure 7a. The proton conductivity test of SPEEK, SPEEK/Pg–C_3_N_4_–1, and SPEEK/Pg–C_3_N_4_–2 membranes did not reach 80 °C because they were already dissolved in water before reaching 80 °C. When the Pg–C_3_N_4_ addition amount was below 3%, the proton conductivity of the membrane increased with the increase in the Pg–C_3_N_4_ addition amount. The improvement of proton conductivity was owing to two main reasons: (1) the abundant –NH– and –NH_2_ groups on Pg–C_3_N_4_ nanosheets form acid–base pairs with the –SO_3_H groups in SPEEK, which increases the transfer of protons; and (2) the Pg–C_3_N_4_ nanosheets have a high aspect ratio, which can guide the uniform arrangement and interconnection of ion clusters in the SPEEK polymer, thus constructing a continuous proton transfer channel. The proton conductivity at 80 °C and 100% RH drops from 138 to 121 mS/cm when the added amount of Pg–C_3_N_4_ increases from 3% to 4%. This is because the aggregation of Pg–C_3_N_4_ may block the channels for proton conduction.

The activation energy of proton transfer in the membrane was calculated by the Arrhenius formula, as shown in Figure 7b. The activation energy of proton transfer in SPEEK, SPEEK/Pg–C_3_N_4_–1, SPEEK/Pg–C_3_N_4_–2, SPEEK/Pg–C_3_N_4_–3, and SPEEK/Pg–C_3_N_4_–4 membranes was 14.9, 14.5, 12.3, 10.1, and 11.1 kJ·mol^−1^, respectively, confirming that protons in the polymer quantum dot membrane were mainly transferred through the Grotthuss mechanism [20,21]. Compared with the SPEEK composite membrane materials reported in the previous literature, the SPEEK/Pg–C_3_N_4_–3 membrane prepared in this study has better comprehensive performance (Figure 7c and Table 1).

The fuel cell performance of SPEEK and SPEEK/Pg–C_3_N_4_–3 membranes is shown in Figure 7d. The open circuit voltage of the fuel cell reached 0.954 V, which is higher than the 0.94 V of SPEEK, indicating that the SPEEK/Pg–C_3_N_4_–3 membrane has low gas permeability. Compared with the SPEEK membrane, the fuel cell performance of the SPEEK/Pg–C_3_N_4_–3 membrane was 21% higher, thanks to its higher proton conductivity, which reduced the internal resistance of the fuel cell and improved its output power density. The output power density of the SPEEK/Pg–C_3_N_4_–3 membrane fuel cell reached a maximum of 326.8 mW/cm^2^, while the performance of Nafion 117 and Nafion 115 membrane materials measured under similar conditions was 313.6 mW/cm^2^ (75 °C and 95% RH) and 216.5 mW/cm^2^ (80 °C and 100% RH), respectively [34,35,36]. The results demonstrate the potential of composite SPEEK membranes in fuel cell applications.

## 4. Conclusions

In this study, porous graphitic C_3_N_4_ (Pg–C_3_N_4_) nanosheets were added to SPEEK polymer to prepare composite membranes. Because the –NH_2_ and –NH– groups on the porous C_3_N_4_ nanosheets could interact with the sulfonic acid groups in SPEEK and form a more continuous proton transfer channel, the proton conductivity of the SPEEK composite membranes with 3% C_3_N_4_ nanosheet addition (SPEEK/Pg–C_3_N_4_–3) membrane achieved 138 mS/cm^−1^, 50% higher than the ordinary SPEEK membrane. Furthermore, the interfacial interaction between the nanosheets and the polymer chains can reduce the movement of the polymer; therefore, the SPEEK/Pg–C_3_N_4_–3 membrane can remain stable in water at 80 °C and has a mechanical strength of 74.1 MPa. The power density of the fuel cell using the SPEEK/Pg–C_3_N_4_–3 membrane reached 326.8 mW/cm^−2^, which was 21% higher than that of the fuel cell using the ordinary SPEEK membrane. This study provides a new and simple method to break through the trade–off phenomenon between the proton conductivity and membrane strength of the SPEEK membrane.

## Figures and Tables

**Table 1:** Proton conductivity and tensile strength of the prepared membranes in comparison with SPEEK–based membranes.

Membrane	Temperature (°C)	RH (%)	Proton Conductivity (mS cm^−1^)	Tensile Strength (MPa)	Ref.
SPEEK	60	100	90	41.9	This work
SPEEK/Pg–C_3_N_4_–3	80	100	138	74.1	This work
SPEEK/clay–SO_3_H–1	80	100	166	48.3	[22]
SPKCL–1	80	/	18.4	48.7	[23]
SPEEK–90/PAI–10/S–SiO_2_–3	81.2	/	81.2	42	[24]
SPEEK–80/SPVDF–HFP–20/S–SiO_2_–6	90	/	79	38.5	[25]
SPEEK/TiNFs–1.0	20	/	102.6	40.4	[26]
SPPEK/SZrTi–10.12	120	100	29.21	69	[27]
SPPEK/TiO_2_@CNT–5	80	/	104	47.1	[28]
SPEEK/ZCN–2.5	120	30	50.24	50.8	[29]
SPEEK/ZIF–COOH–5	80	/	15.2	44.1	[30]
SPEEK/GO–his–4	75	100	290	38.8	[11]
SP–SG–5	/	/	47	35.7	[31]
SPEEK/CN–0.5	55	100	183	51.31	[15]
SPEEK/HPW/g–C_3_N_4_–1.0	60	100	52.1	51.1	[14]
S/CNT@PDA–3%	/	/	97.7	39.4	[32]
SPEEK/PABS–SWCNT	80	/	61.1	25.6	[33]

## Data Availability

Data are contained within the article.

## Supplementary Materials

The following supporting information can be downloaded at: https://www.mdpi.com/article/10.3390/membranes15070194/s1, Figure S1: SEM (a) and AFM (b) image of Pg–C_3_N_4_ nanosheets.
