# Supplementary material for: Simultaneously Promoting Proton Conductivity and Mechanical Stability of SPEEK Membrane by Incorporating Porous g–C3N4
Source: Membranes (Basel). 2025 Jun 29;15(7):194. doi: 10.3390/membranes15070194 (PMC12299626; doi:10.3390/membranes15070194)
Supplement: Supplementary file 1 [file membranes-15-00194-s001.zip › membranes-3675901-supplementary.pdf]

Supporting Information

Simultaneously promoting proton conductivity and mechanical stability of SPEEK membrane by incorporating porous g-C_3N_4

Xiaoyao Wang [1,2,*] and Benbing Shi[3,4*]

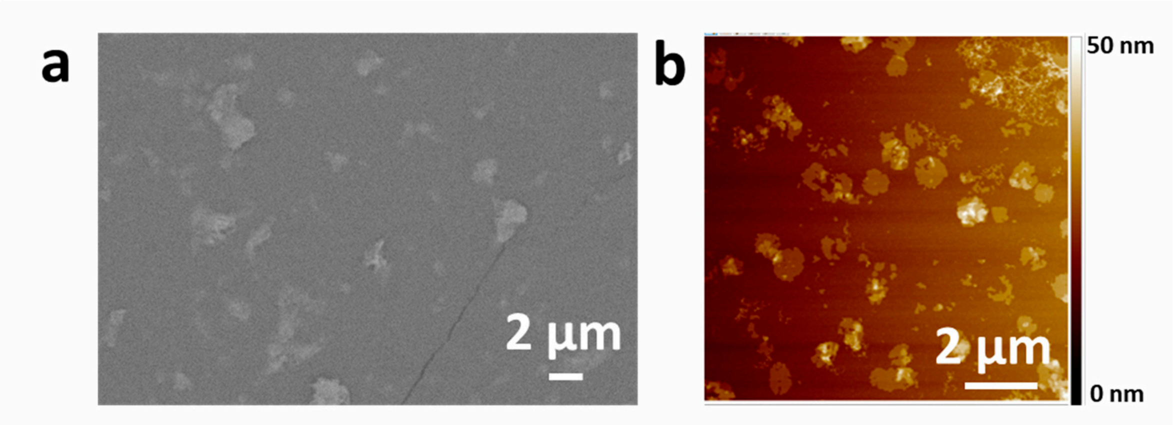

Figure S1. SEM (a) and AFM (b) image of Pg-C_3N_4 nanosheets
